# Antihyperlipidemic and Antioxidant Potential of *Paeonia emodi* Royle against High-Fat Diet Induced Oxidative Stress

**DOI:** 10.1155/2014/182362

**Published:** 2014-03-10

**Authors:** Bilal A. Zargar, Mubashir H. Masoodi, Bahar Ahmed, Showkat A. Ganie

**Affiliations:** ^1^Department of Pharmaceutical Sciences, University of Kashmir, Hazratbal, Srinagar, Jammu and Kashmir 190006, India; ^2^Department of Pharmaceutical Chemistry, Faculty of Pharmacy, Jamia Hamdard University, New Delhi 110062, India; ^3^Department of Clinical Biochemistry, University of Kashmir, Hazratbal, Srinagar, Jammu and Kashmir 190006, India

## Abstract

The present study was intended to evaluate the effects of *Paeonia emodi* rhizome extracts on serum triglycerides (TGs), total cholesterol (TC), low density lipoprotein cholesterol (LDL-c), high density lipoprotein cholesterol (HDL-c), atherogenic index (AI), superoxide dismutase (SOD), and glutathione peroxidase (GPx). The plant was extensively examined for its *in vitro* antioxidant activity, and the preliminary phytochemical screening was carried out using standard protocols. Male Wistar rats were induced with hyperlipidemia using high-fat diet and were treated orally with hydroalcoholic and aqueous extracts at the dose of 200 mg/kg bw for 30 days. TGs, TC, LDL-c, and AI were significantly reduced while HDL-c, SOD, and GPx levels rose to a considerable extent. After subjecting to acute toxicity testing, the extracts were found to be safe. The observations suggest antihyperlipidemic and antioxidant potential of *P. emodi* in high-fat diet induced hyperlipidemic/oxidative stressed rats.

## 1. Introduction

Reactive oxygen species (ROS) comprises superoxide, hydroxyl, peroxyl (RO_2_^∙^), alkoxyl (RO^∙^), and hydroperoxyl (HO_2_^∙^) radicals. Nitric oxide and nitrogen dioxide (^∙^NO_2_) are two nitrogen free radicals. Oxygen and nitrogen free radicals can be converted to other nonradical reactive species, such as hydrogen peroxide, hypochlorous acid (HOCl), and peroxynitrite (ONOO^−^). ROS, reactive nitrogen species (RNS), and reactive chlorine species are generated in aerobic cells under physiologic and pathologic conditions [1]. Thus, ROS and RNS include radical and nonradical species. These species are maintained at very low steady-state concentration by the antioxidant system, but when their production increases they may overcome the scavenger capacity of the antioxidant system, resulting in an oxidative stress and damage to biological targets. Plasma lipoproteins are protected against oxidative modification by the antioxidant defense system of the organism. This system is constituted by the enzymes like SOD, glutathione peroxidase (GPx), and catalase, as well as hydrophilic antioxidants such as ascorbate, reduced glutathione, and urate. Lipoprotein particles carry lipophilic antioxidants, such as tocopherols and carotenoids. All of these interact with free radicals or block free radical chain reactions [2].

Endothelial cells, smooth muscle cells, and macrophages are the sources of oxidants for the oxidative modification of phospholipids. Ox-LDL can damage endothelial cells and induce the expression of adhesion molecules such as P-selectin and chemotactic factors such as monocyte chemoattractant protein-1 and macrophage colony stimulating factor. These processes lead to the tethering, activation, and attachment of monocytes and T lymphocytes to the endothelial cells [3]. There is evidence that macrophages in atherosclerotic lesions express myeloperoxidase that yields a unique pattern of protein oxidation products. Myeloperoxidase is also pinpointed as a pathway that promotes LDL oxidation [4]. Natural products with antioxidant potential have been proposed to be effective in the treatment of metabolic syndrome which may ultimately be used in the treatment of insulin resistance, diabetes, obesity, altered lipid profile, and hypertension [5–7].

*Paeonia emodi *has widely been used in the indigenous and traditional systems of medicine because of its wide therapeutic profile. The fleshy roots are used in uterine diseases, biliousness, dropsy, and nervous affections; they are also prescribed as a blood purifier for children. Excessive doses cause headache, confused vision, and vomiting. The seeds are emetic and cathartic. An infusion of the dried flowers is given to control diarrhea. The rhizome of* P. emodi* has been indicated for the treatment of headache, abdominal spasms, and hysteria and is also used as nervine tonic. It is one of the constituents of important formulation in Unani pharmacopoeia which is widely prescribed for the treatment of urinary incontinence. Peony has also been used in different cardiovascular and respiratory illnesses including palpitations, high blood pressure, congestive heart failure, and atherosclerosis [8].

## 2. Materials and Methods

### 2.1. Plant Materials

The plant material consisted of the dried rhizome of* P. emodi *which was collected from Pahalgam area of Jammu and Kashmir during the month of May 2012, authenticated by the curator, and a voucher specimen (KASH-bot/Ku/PH-1201) deposited in the Centre for Plant Taxonomy (KASH), University of Kashmir.

### 2.2. Chemicals and Reagents

Catechin, DPPH, and enzyme kits were purchased from Sigma-Aldrich Chemie GmbH, Industriestrasse 25, Postfach, CH-9471, Buchs, Switzerland. Cholesterol was purchased from Merck KGaA, Darmstadt, Germany. All other reagents and chemicals used were of analytical grade and obtained from HiMedia, A-516, Swastik Disha Business Park, LBS Marg, Mumbai 400 086, India.

### 2.3. Animals

All experiments were performed in compliance with the Indian legislation on the use and care of laboratory animals and were approved by the Institutional Animal Ethics committee (no. KU/Pharm/CPCSEA/2009-01), University of Kashmir. Male Wistar rats (200 ± 50 g) were purchased from the Indian Institute for Integrative Medicine (IIIM), Jammu, India, and they were group-housed (6 rats per cage) with free access to food and water and kept in a regulated environment 25 ± 1°C under 12/12 h (light/dark) cycle.

After 1 week of acclimation to the laboratory, rats were selected randomly into 6 groups (6 animals in each group). The extracts were suspended in normal saline at a dose level of 200 mg/kg body weight. The final volume of extract at each dose was 1 mL which was fed to rats by gavage. Group I received vehicle only at 5 mL/kg/day (NC); group II received high-fat diet (86.8% basic diet, 8% butter, 5% cholesterol, and 0.2% sodium cholate) only (HC); group III were administered high-fat diet along with lovastatin (10 mg/kg/day) (HC/LOVA); group IV received high-fat diet and 1 mL of hydroalcoholic extract of* P. emodi* (HC/HAPE); group V received 1 mL of aqueous extract of* P. emodi* (HC/AQPE). The experiment continued for a period of 5 weeks. Rats were provided free access to food and water during the experimental period. Food consumption and body weight were recorded daily. At the end of the experimental period, all animals were fasted 14 h before anesthetizing with chloral hydrate and sacrificed. Blood samples were drawn from the ophthalmic venous plexus. After centrifugation (5000 g, 15 min, and 4°C), the serum samples were collected and stored at −20°C.

### 2.4. Extract Preparation

Rhizomes of* P. emodi* were washed, shade dried, powdered in a heavy-duty Willy mill (Bells India Ltd.), and then soaked in 70% ethanol overnight. After filtration, the residue was then subjected to two more subsequent extractions. The filtrates obtained were combined and the solvent was removed in a rotary vacuum evaporator at 40°C for obtaining hydroalcoholic extract. The same procedure was followed for the preparation of aqueous extract.

### 2.5. Acute Oral Toxicity Testing

Acute toxicity study was carried out* in vivo* in the albino rats. Solutions of the dried extracts were prepared using normal saline. The study was conducted as per Organization of Economic Cooperation and Development (OECD) Test guidelines on acute oral toxicity under a computer-guided statistical programme (AOT425StatPgm, version 1.0). Up and down procedure was conducted, using the dose increment of 175 mg/kg p.o., 550 mg/kg p.o., and 2000 mg/kg p.o. of the aqueous and hydroalcoholic extracts (acute oral toxicity (AOT) (OECD Test guideline 425) statistical programme (AOT425StatPgm), 2001).

### 2.6. Phytochemical Screening of the Crude Extracts

The hydroalcoholic and aqueous* P. emodi* rhizome extracts were subjected to phytochemical screening for the detection of various phytoconstituents [9, 10].

### 2.7. Total Phenolic Content (TPC)

The total phenolic content of HAPE and AQPE was determined by Folin-Ciocalteu reagent according to the method of Singleton, Orthofer, and Lamuela-Raventos (1999). Results were expressed as mg gallic acid equivalents per g extract [11].

### 2.8. *In Vitro* Antioxidant Studies

#### 2.8.1. DPPH Radical Scavenging Activity

The assay was conducted on the basis of scavenging activity of the stable DPPH free radical following the method described by Blois with some minor modifications [12, 13]. To 1 mL of the sample (10, 20, 30, 40, and 50 *μ*g/mL) was added 3 mL of a 0.1 mmol/L methanol solution of DPPH. The absorbance of all the samples was determined at 517 nm (UVD 2960, Labomed, Inc.) after an incubation period of 30 minutes. The percentage inhibitory activity was determined according to the following formula:

(1) $$\begin{array}{c} \%\;\text{inhibition}=\left[1\;-\;\left(\frac{A_{e}}{A_{0}}\right)\right]\times 100, \end{array}$$

where *A*_0_ is the absorbance without sample and *A*_*e*_ is the absorbance with sample.

#### 2.8.2. Reducing Power

The assay was conducted according to the method of Oyaizu [14]. According to this method, the reduction of Fe^3+^ to Fe^2+^ was determined by measuring absorbance of Pearl's Prussian blue complex. This method is based on the reduction of (Fe^3+^) ferricyanide in stoichiometric excess relative to the antioxidants. To different concentrations of the extracts (10, 20, 30, 40, and 50 *μ*g/mL) were added 2.5 mL of 0.2 M phosphate buffer (pH 6.6) and 2.5 mL of 1% potassium ferricyanide [K_3_Fe(CN)_6_]. The mixture was incubated at 50°C for 20 min and 2.5 mL of 10% trichloroacetic acid was added to the mixture and centrifuged at 3000 rpm for 10 min. The upper layer (2.5 mL) was added to (2.5 mL) distilled water and FeCl_3_ (0.5 mL, 0.1%), and the absorbance was measured at 700 nm. BHT was used as the standard and the percentage reduction was calculated using the following formula:

(2) $$\begin{array}{c} \%\;\text{reduction}=\left[1\;-\;\left(1-\;\frac{A_{c}}{A_{s}}\right)\right]\times 100. \end{array}$$

#### 2.8.3. Hydroxyl Radical Scavenging

The assay was conducted according to the method of Halliwell et al. [15]. It is based on the measurement of thiobarbituric acid reactive species (predominantly malondialdehyde producing pink color on reaction with thiobarbituric acid) generated from the degradation of deoxyribose on exposure to hydroxyl radical. Hydroxyl radical is generated from Fe^3+^-ascorbate-H_2_O_2_ system (Fenton reaction).

The reaction mixture contains 25 mM deoxyribose, 10 mM ferric chloride, 100 mM ascorbic acid, 2.8 mM H_2_O_2_ in 10 mM KH_2_PO_4_ (pH 7.4), and various concentrations of plant extracts (10–50 *μ*g/mL). The reaction mixture was incubated at 37°C for 1 h. 1% thiobarbituric and 3% trichloroacetic acid (1 mL each) were added, and the mixture was heated at 100°C for 20 min. The gradation in color intensity was measured spectrophotometrically at 532 nm. The results were expressed as percentage inhibition of deoxyribose oxidation using the formula below:

(3) $$\begin{array}{c} \%\;\text{inhibition}=\left(A_{c}-\frac{A}{A_{c}}\right)\times 100, \end{array}$$

where *A*_*c*_ is the absorbance in the presence of control and *A* is the absorbance in the presence of the extract.

#### 2.8.4. Superoxide Radical Scavenging Activity

The assay was based on the capacity of the extracts to inhibit formazan formation by scavenging the superoxide radicals generated in riboflavin-light-NBT system [16]. The reaction mixture contained 50 mM phosphate buffer (pH 7.6), 20 *μ*g riboflavin, 12 mM EDTA, and NBT 0.1 mg/3 mL, added in sequence. Reaction was started by illuminating the reaction mixture with different concentrations of sample extract/standard for 90 seconds. Immediately after illumination, the absorbance was measured at 590 nm. Rutin was used as positive control. The percentage of superoxide anion scavenged was calculated by using the following equation:

(4) $$\begin{array}{c} \%\;\text{inhibition}=\left(1-\;\frac{A_{s}}{A_{c}}\right)\times 100. \end{array}$$

#### 2.8.5. H_2_O_2_ Scavenging Activity

The hydrogen peroxide scavenging assay was carried out following the procedure of Ruch et al. [17]. The principle of this method is that there is a decrease in absorbance of H_2_O_2_ upon oxidation of H_2_O_2_. A solution of 43 mM H_2_O_2_ was prepared in 0.1 M phosphate buffer (pH 7.4). 1 mL extract (each at different concentrations) in 3.4 mL phosphate buffer was added to 0.6 mL of H_2_O_2_ solution (43 mM), and absorbance of the reaction mixture was recorded at 230 nm. A blank solution contained the sodium phosphate buffer without H_2_O_2_.

The percentage of H_2_O_2_ scavenging by the extracts and standard was calculated using the following equation:

(5) $$\begin{array}{c} \%\;\text{inhibition}=\left(1-\;\frac{A_{s}}{A_{c}}\right)\times 100, \end{array}$$

where *A*_*c*_ is the absorbance of the control and *A*_*s*_ is the absorbance in the presence of extracts.

### 2.9. *In Vivo* Studies

#### 2.9.1. Lipid Profile

Blood samples were collected and centrifuged at 3500 g for 15 min to obtain serum. The levels of serum total cholesterol (TC), triglycerides (TG), and high density lipoprotein cholesterol (HDL-c) were determined using commercially available kits (purchased from Sigma-Aldrich Chemie GmbH, Industriestrasse 25, Postfach, CH-9471, Buchs, Switzerland) according to the manufacturers' instructions. Low density lipoprotein cholesterol (LDL-c) was calculated according to the Friedwald equation:

(6) $$\begin{array}{c} \left[\text{LDLc}\right]=\left[\text{Total}\;\text{cholesterol}\right]-\left[\text{HDLc}\right]-\frac{\left[\text{TG}\right]}{5}. \end{array}$$

The arteriosclerosis index (AI) was calculated by the equations as follows:

(7) $$\begin{array}{c} \text{AI}=\text{Tc}-\frac{\text{HDLc}}{\text{HDLc}}. \end{array}$$

#### 2.9.2. Glutathione Peroxidase (GPx)

Glutathione peroxidase activity was assayed using the method of Rotruck et al., 1973 [18].

#### 2.9.3. Superoxide Dismutase (SOD)

Superoxide dismutase (SOD) activity was estimated by the method of S. Marklund and G. Marklund, 1974 [19]. The reaction was maintained at pH 7.9 and is based on the ability of the enzyme to inhibit the autoxidation of pyrogallol.

### 2.10. Statistical Analyses

The data has been analysed using descriptive statistics, *t*-test, and one way ANOVA followed by post hoc analysis (Dunnett's test), using SPSS-20. Statistical significance was considered at *P* < 0.05.

## 3. Results

### 3.1. Acute Oral Toxicity Study

The hydroalcoholic as well as aqueous extracts did not cause any mortality up to 2000 mg/Kg body weight and were considered safe.

### 3.2. Phytochemical Investigation

Qualitative phytochemical screening revealed that the rhizomes of* P. emodi* contain carbohydrates, reducing sugars, phenolics and tannins, cardiac glycosides, anthraquinone glycosides, terpenes and steroids, and resins and oxalic, tartaric, citric, and ascorbic acids.

### 3.3. Total Phenolic Content

Total phenolic content of the hydroalcoholic extract was found to be 375.83 ± 3.82 and that of aqueous extract 187.83 ± 2.52. The phenolic contents in the samples were expressed as mg of gallic acid equivalent (GAE) for every g of sample (mg GAE/g).

### 3.4. DPPH Radical Scavenging

**Figure 1:**
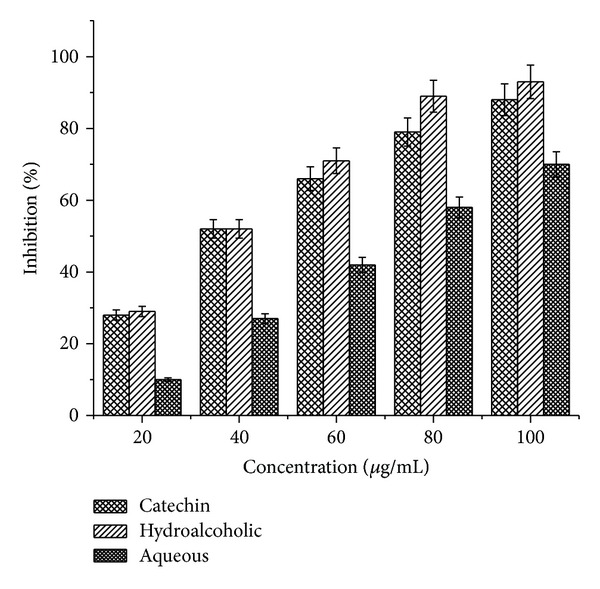
DPPH radical scavenging activity of hydroalcoholic and aqueous extracts of* P. emodi *Royle.

Both extracts showed a concentration dependent scavenging of DPPH radicals as shown in Figure 1. The scavenging effect of the hydroalcoholic and aqueous extract was 93% and 70%, respectively, at a concentration of 100 *μ*g/mL. At the same concentration the scavenging activity of catechin (a known antioxidant) was highly comparable.

### 3.5. Reducing Power

**Figure 2:**
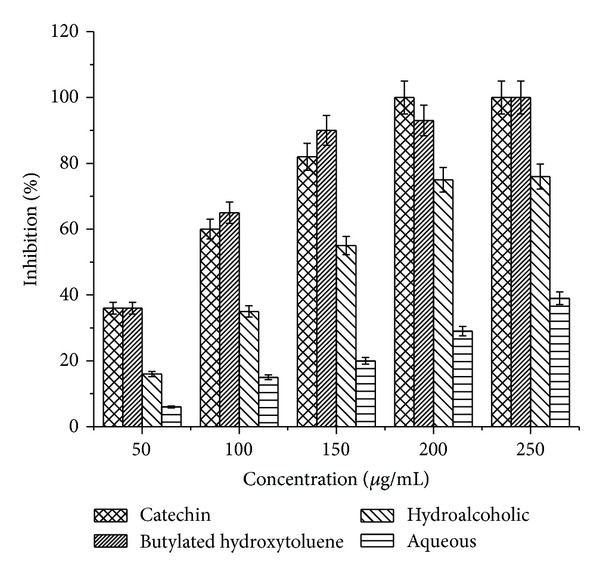
Reducing power of hydroalcoholic and aqueous extracts of* P. emodi *Royle.

In the present study, it was found that the reducing power of the plant extracts and known antioxidants (catechin and BHT) on Fe^3+^ was concentration dependent. The reducing power increased with increasing concentration of hydroalcoholic and aqueous extracts (Figure 2). A comparable effect was seen when catechin and BHT were used as positive control.

### 3.6. Hydroxyl Radical Scavenging

**Figure 3:**
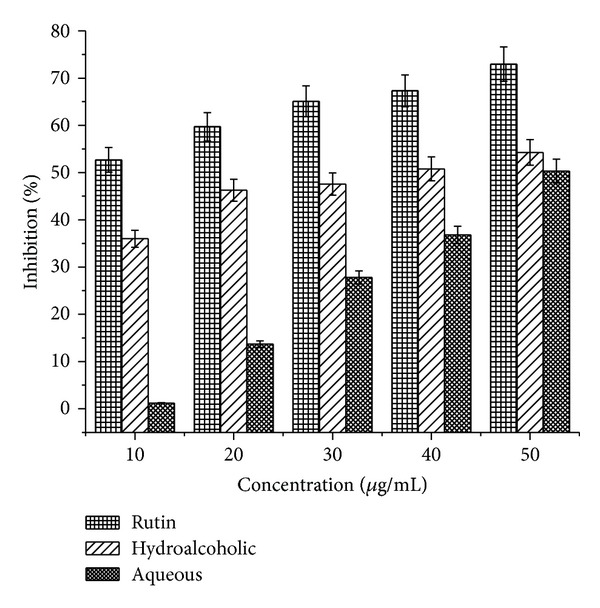
Hydroxyl radical scavenging activity of hydroalcoholic and aqueous extracts of* P. emodi *Royle.

As can be seen from Figure 3, both the extracts show a concentration dependent scavenging of hydroxyl radical. The hydroalcoholic and aqueous extracts showed a 54.3% and 50.3% scavenging effect at a concentration of 50 *μ*g/mL on hydroxyl radicals which was comparable to that of rutin, a known antioxidant.

### 3.7. Superoxide Radical Scavenging

**Figure 4:**
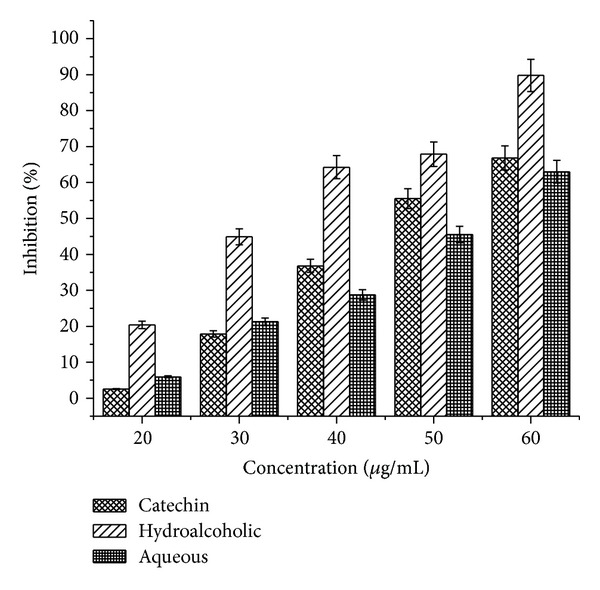
Superoxide radical scavenging activity of hydroalcoholic and aqueous extracts of* P. emodi *Royle.

Both the tested extracts exhibited effective O_2_^−∙^ scavenging activity. As evident from Figure 4, both the extracts were found to be effective superoxide radical scavengers in a concentration dependent manner (20−60 *μ*g/mL). The highest activity was shown by hydroalcoholic extract followed by catechin and aqueous extract.

### 3.8. Effect on H_2_O_2_ Scavenging

**Figure 5:**
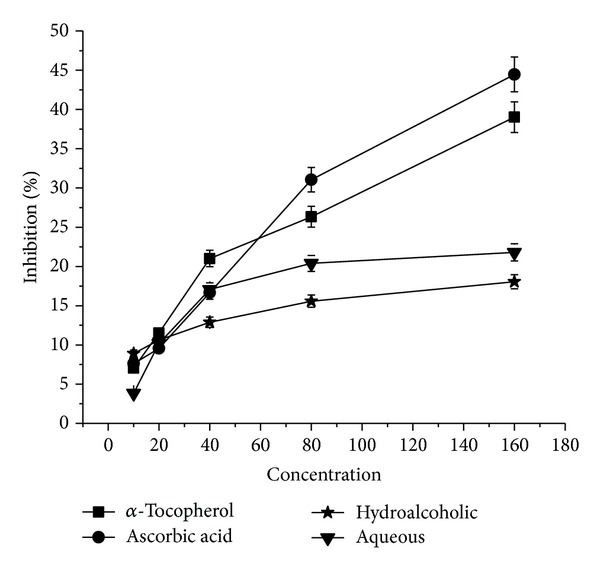
Hydrogen peroxide scavenging activity of hydroalcoholic and aqueous extracts of* P. emodi *Royle.

As evident from Figure 5, both the extracts have been found to be poor H_2_O_2_ scavengers compared to that of standard antioxidants *α*-tocopherol and ascorbic acid.

### 3.9. *In Vivo* Lipid Parameters

Male Wistar rats fed with high-fat diet showed a marked elevation in serum triglycerides, total, and LDL cholesterol. Administration of hydroalcoholic and aqueous extracts as well as lovastatin significantly restored the elevated lipids to a considerable extent as shown in Table 1 (*P* < 0.05). The group administered with aqueous extract showed HDL increase (considered to be good cholesterol) to a much larger extent.

### 3.10. Effect on Serum Antioxidant Enzymes

Significant reduction (*P* < 0.05) in the antioxidant enzyme activities was observed in hyperlipidemic rats and depicted in Table 1. When hydroalcoholic and aqueous extracts were administered to hyperlipidemic rats, restoration in serum SOD and GPx was found to a great extent.

## 4. Discussion

The rhizome extracts of* P. emodi* have been found to be safe, without any acute or long-term toxic effect. The hydroalcoholic and aqueous extracts demonstrated promising antioxidant activities both* in vitro* and* in vivo*. Both the extracts were found to have a concentration dependent increase in their antioxidant potentials with varying degrees of efficiencies. The present study demonstrates that the extracts contain polyphenolics as potent antioxidants which have the ability to scavenge DPPH^∙^, O_2_^−∙^, and H_2_O_2_ and reduce Fe^3+^ (ferricyanide complex) to the ferrous form. The Fe^2+^ formed can be monitored by measuring the formation of Pearl's Prussian blue at 700 nm.

Superoxide is an oxygen-centered radical with selective reactivity. Although it is a relatively weak oxidant, superoxide exhibits limited chemical reactivity but can generate more dangerous species, including singlet oxygen and hydroxyl radicals, which cause the peroxidation of lipids [20]. These species are produced by a number of enzyme systems. Superoxide can also reduce certain iron complexes such as cytochrome c. Superoxide anions are thus precursors to active free radicals that have potential for reacting with biological macromolecules and thereby inducing tissue damage [21]. Also, superoxide has been observed to directly initiate lipid peroxidation. It has also been reported that antioxidant properties of some flavonoids are effective mainly via scavenging of superoxide anion radical [22]. Superoxide radicals are normally formed first, and their effects can be magnified because they produce other kinds of free radicals and oxidizing agents [23]. Superoxide anions derived from dissolved oxygen by the riboflavin-light-NBT system will reduce NBT in this system. In this method, superoxide anion reduces the yellow dye (NBT^2+^) to produce the formazan, which is measured spectrophotometrically at 590 nm. Antioxidants are able to inhibit the blue NBT formation [24]. The decrease of absorbance at 560 nm with antioxidants indicates the consumption of superoxide anion in the reaction mixture. Figure 4 shows the inhibition of superoxide radical generation by different concentrations of extracts and standards.

The extracts were also found to be viable scavengers of hydroxyl radical which is involved in DNA cross-links and strand-breaks and is considered to be one of the quick initiators of lipid peroxidation [25]. The ability of the extracts to scavenge ^∙^OH can be related to the prevention of lipid peroxidation. It can be inferred that the extracts might prevent reactive radical species from damaging biomolecules such as lipoproteins, polyunsaturated fatty acids, DNA, amino acids, proteins, and sugars in biological systems [26]. In this study, the antioxidant activity was compared to catechin, ascorbic acid, *α*-tocopherol, and BHT.

The present study demonstrates that the extracts were able to reduce both triglycerides and LDL cholesterol to significant levels which is very encouraging. The aqueous extract has additionally shown an enormous increase in the levels of HDL cholesterol.

Hyperlipidemia causes oxidative stress and reduces the oxidant defense system, thereby elevating the lipid peroxides. Free radical scavenging enzymes such as SOD, CAT, and GPx are the first line of cellular defense against oxidative injury and are involved in the disposal of superoxide anions, hydrogen peroxide, and so forth, and pronounced alterations were observed during hyperlipidemic conditions in rats [27]. The increased SOD levels after administration of the extracts can be attributed to the large amounts of ascorbic acid present in the plant which was confirmed during the preliminary phytochemical screening. There are two forms of the enzyme glutathione peroxidase, one of which is selenium-independent (glutathione-S-transferase, GST, EC 2.5.1.18) while the other is selenium-dependent (GPx, EC 1.11.1.19) [18]. These two enzymes differ in the number of subunits, the bonding nature of the selenium at the active centre, and their catalytic mechanisms. Glutathione metabolism is one of the most essential of antioxidative defense mechanisms. Humans have four different Se-dependent glutathione peroxidases [28]. All GPx enzymes are known to add two electrons to reduce peroxides by forming selenoles (Se–OH). The antioxidant properties of these selenoenzymes allow them to eliminate peroxides as potential substrates for the Fenton reaction. GPx acts in conjunction with the tripeptide glutathione (GSH), which is present in cells in high (micromolar) concentrations. The substrate for the catalytic reaction of GPx is H_2_O_2_, or organic peroxide ROOH. GPx decomposes peroxides to water (or alcohol) while simultaneously oxidizing GSH : GPx

(8) $$\begin{array}{c} 2\text{GSH}+{\text{H}}_{2}{\text{O}}_{2}\overset{\text{GPx}}{⟶}\text{GSSH}+2{\text{H}}_{2}\text{O}\\ 2\text{GSH}+\text{ROOH}\overset{\text{GPx}}{⟶}\text{GSSH}+\text{ROH}+{\text{H}}_{2}\text{O} \end{array}$$

Significantly, GPx competes with catalase for H_2_O_2_ as a substrate and is the major source of protection against low levels of oxidative stress. Table 1 clearly indicates that there is a considerable decrease in the levels of GPx in negative control group while there is a restoration of the enzyme levels in the groups fed with plant extracts.

## Figures and Tables

**Table 1:** Effect of hydroalcoholic and aqueous root extract of *P. emodi* on lipid profile, atherogenic index, and serum antioxidant enzyme status in male Wistar rats.

Parameters	NC	HC	HC/LOVA	HC/HAPE	HC/AQPE
TG	39.18 ± 1.08	73.39 ± 4.24	49.80 ± 2.12	39.31 ± 2.87	52.12 ± 1.26
TC	58.14 ± 2.51	69.65 ± 2.35	53.73 ± 1.27	47.35 ± 3.00	54.89 ± 2.24
HDL	27.55 ± 2.14	18.41 ± 0.74	27.73 ± 1.66	24.55 ± 1.49	35.92 ± 0.95
LDL	13.19	24.24	16.53	13.06	8.55
AI	57.14	68.65	52.73	46.35	53.89
GPx	12.13 ± 1.21	5.22 ± 0.92	8.13 ± 1.03	11.83 ± 1.05	11.27 ± 1.98
SOD	10.92 ± 1.12	4.31 ± 1.11	6.71 ± 0.87	9.18 ± 1.82	9.07 ± 1.34

Values are expressed as mean ± SEM. At *P* < 0.05, results were found to be significantly different from control group. NC: normal control, HC: hyperlipidemic control, LOVA: lovastatin, HAPE: hydroalcoholic extract of *P. emodi*, and AQPE: aqueous extract of *P. emodi*.

SOD is expressed as U/mg of protein while GPx is expressed as *μ*g of GSH utilized/min/mg protein.

## References

[1] Evans P, Halliwell B (2001). Micronutrients: oxidant/antioxidant status. *British Journal of Nutrition*.

[2] Araujo FB, Barbosa DS, Hsin CY, Maranhao RC, Abdalla DSP (1995). Evaluation of oxidative stress in patients with hyperlipidemia. *Atherosclerosis*.

[3] Madamanchi NR, Vendrov A, Runge MS (2005). Oxidative stress and vascular disease. *Arteriosclerosis, Thrombosis, and Vascular Biology*.

[4] Heinecke JW (2003). Oxidative stress: new approaches to diagnosis and prognosis in atherosclerosis. *The American Journal of Cardiology*.

[5] Iyer A, Panchal S, Poudyal H, Brown L (2009). Potential health benefits of Indian spices in the symptoms of the metabolic syndrome: a review. *Indian Journal of Biochemistry and Biophysics*.

[6] Cui S-C, Yu J, Zhang X-H, Cheng M-Z, Yang L-W, Xu J-Y (2013). Antihyperglycemic and antioxidant activity of water extract from *Anoectochilus roxburghii* in experimental diabetes. *Experimental and Toxicologic Pathology*.

[7] Sancheti S, Sancheti S, Seo S-Y (2013). Antidiabetic and antiacetylcholinesterase effects of ethyl acetate fraction of Chaenomeles sinensis (Thouin) Koehne fruits in streptozotocin-induced diabetic rats. *Experimental and Toxicologic Pathology*.

[8] Zargar BA, Masoodi MH, Ahmed B, Akbar S (2013). *Paeonia emodi* Royle: ethnomedicinal uses, phytochemistry and pharmacology. *Phytochemistry Letters*.

[9] Harborne JB (1976). *Phytochemical Methods to Modern Techniques of Plant Analysis*.

[10] Wagner H, Bladt S, Zgainski EM (1984). *Plant Drug Analysis*.

[11] Singleton VL, Orthofer R, Lamuela-Raventós RM (1998). Analysis of total phenols and other oxidation substrates and antioxidants by means of folin-ciocalteu reagent. *Methods in Enzymology*.

[12] Blois MS (1958). Antioxidant determinations by the use of a stable free radical. *Nature*.

[13] Ganie SA, Zargar BA, Masood A, Zargar MA (2013). Hepatoprotective and antioxidant activity of rhizome of Podophyllum hexandrum against carbon tetra chloride induced hepatotoxicity in rats. *Biomedical and Environmental Sciences*.

[14] Oyaizu M (1986). Studies on products of browning reaction prepared from glucosamine. *Japanese Journal of Nutrition*.

[15] Halliwell B, Gutteridge JMC, Aruoma OI (1987). The deoxyribose method: a simple “test-tube” assay for determination of rate constants for reactions of hydroxyl radicals. *Analytical Biochemistry*.

[16] Beauchamp C, Fridovich I (1971). Superoxide dismutase: mproved assays and an assay applicable to acrylamide gels. *Analytical Biochemistry*.

[17] Ruch RJ, Cheng S-J, Klaunig JE (1989). Prevention of cytotoxicity and inhibition of intercellular communication by antioxidant catechins isolated from Chinese green tea. *Carcinogenesis*.

[18] Rotruck JT, Pope AL, Ganther HE, Swanson AB, Hafeman DG, Hoekstra WG (1973). Selenium: biochemical role as a component of glatathione peroxidase. *Science*.

[19] Marklund S, Marklund G (1974). Involvement of the superoxide anion radical in the autoxidation of pyrogallol and a convenient assay for superoxide dismutase. *European Journal of Biochemistry*.

[20] Halliwell B, Chirico S, Crawford MA, Bjerve KS, Gey KF (1993). Lipid peroxidation: its mechanism, measurement, and significance. *The American Journal of Clinical Nutrition*.

[21] Halliwell B, Gutteridge JMC (1984). Oxygen toxicity, oxygen radicals, transition metals and disease. *Biochemical Journal*.

[22] Yen G-C, Duh P-D (1994). Scavenging effect of methanolic extracts of peanut hulls on free-radical and active-oxygen species. *Journal of Agricultural and Food Chemistry*.

[23] Liu F, Ooi VEC, Chang ST (1997). Free radical scavenging activities of mushroom polysaccharide extracts. *Life Sciences*.

[24] Parejo I, Viladomat F, Bastida J (2002). Comparison between the radical scavenging activity and antioxidant activity of six distilled and nondistilled mediterranean herbs and aromatic plants. *Journal of Agricultural and Food Chemistry*.

[25] Kappus H (1991). Lipid peroxidation—mechanism and biological relevance. *Free Radicals and Food Additives*.

[26] Halliwell B, Aeschbach R, Löliger J, Aruoma OI (1995). The characterization of antioxidants. *Food and Chemical Toxicology*.

[27] Vijayaraj P, Muthukumar K, Sabarirajan J, Nachiappan V (2013). Antihyperlipidemic activity of Cassia auriculata flowers in triton WR 1339 induced hyperlipidemic rats. *Experimental and Toxicologic Pathology*.

[28] Matés JM, Pérez-Gómez C, De Castro IN (1999). Antioxidant enzymes and human diseases. *Clinical Biochemistry*.

